# The mitochondrial protein TSPO in Alzheimer’s disease: relation to the severity of AD pathology and the neuroinflammatory environment

**DOI:** 10.1186/s12974-023-02869-9

**Published:** 2023-08-14

**Authors:** Emma F. Garland, Oliver Dennett, Laurie C. Lau, David S. Chatelet, Michel Bottlaender, James A. R. Nicoll, Delphine Boche

**Affiliations:** 1 Clinical Neurosciences, Clinical and Experimental Sciences, Faculty of Medicine, University of Southampton, Southampton General Hospital, Southampton, SO16 6YD UK; 2 Biomedical Imaging Unit, University of Southampton, Southampton General Hospital, Southampton, SO16 6YD UK; 3 CEA, CNRS, Inserm, BioMaps, Service Hospitalier Frederic Joliot, Paris-Sacaly University, 91400 Orsay, France; 4 UNIACT Neurospin, CEA, Gif-Sur-Yvette, 91191 France; 5 Department of Cellular Pathology, University Hospital Southampton NHS Trust, Southampton, SO16 6YD UK

**Keywords:** TSPO, Alzheimer’s disease, Cerebellum, Pathology, Inflammation, Microglia, Human post-mortem

## Abstract

The 18kD translocator protein (TSPO) is used as a positron emission tomography (PET) target to quantify neuroinflammation in patients. In Alzheimer’s disease (AD), the cerebellum is the pseudo-reference region for comparison with the cerebral cortex due to the absence of AD pathology and lower levels of TSPO. However, using the cerebellum as a pseudo-reference region is debated, with other brain regions suggested as more suitable. This paper aimed to establish the neuroinflammatory differences between the temporal cortex and cerebellar cortex, including TSPO expression. Using 60 human *post-mortem* samples encompassing the spectrum of Braak stages (I–VI), immunostaining for pan-Aβ, hyperphosphorylated (p)Tau, TSPO and microglial proteins Iba1, HLA–DR and MSR-A was performed in the temporal cortex and cerebellum. In the cerebellum, Aβ but not pTau, increased over the course of the disease, in contrast to the temporal cortex, where both proteins were significantly increased. TSPO increased in the temporal cortex, more than twofold in the later stages of AD compared to the early stages, but not in the cerebellum. Conversely, Iba1 increased in the cerebellum, but not in the temporal cortex. TSPO was associated with pTau in the temporal cortex, suggesting that TSPO positive microglia may be reacting to pTau itself and/or neurodegeneration at later stages of AD. Furthermore, the neuroinflammatory microenvironment was examined, using MesoScale Discovery assays, and IL15 only was significantly increased in the temporal cortex. Together this data suggests that the cerebellum maintains a more homeostatic environment compared to the temporal cortex, with a consistent TSPO expression, supporting its use as a pseudo-reference region for quantification in TSPO PET scans.

**Supplementary Information:**

The online version contains supplementary material available at 10.1186/s12974-023-02869-9.

## Introduction

Neuroinflammation, as defined by the reactivity of microglia, has emerged as a key element of the pathogenesis of Alzheimer’s disease (AD) based on genetic findings [1, 2]. Consequently, the need for new methodologies to assess and follow microglial activation in living patients prompted the development of positron emission tomography (PET) ligands for molecular imaging of neuroinflammation [3, 4]. Indeed, it is unclear to what extent microglia, the main immune cells in the brain, promote or respond to neurodegeneration. There is still not a complete understanding as to how microglia participate in the onset and progression of the disease, with the brain environment revealing inflammatory heterogeneity and a mixture of pro- and anti-inflammatory compounds observed *post-mortem* in late stages of the disease [5, 6]. The immune reactions are clearly complex in AD, with evidence of temporal changes of microglia [7], emphasizing the importance of brain imaging in living patients [8].

Molecular imaging studies in AD have focused on visualising activated microglia, most commonly measured by elevated expression of translocator protein 18 kDa (TSPO), a five transmembrane domain protein mainly located in the outer membrane of microglial mitochondria [9–11]. One of the limitations associated with the use of TSPO is its inability to distinguish between the different phenotypes expressed by microglia and potentially lacking specificity [12]. The literature shows divergent and sometimes conflicting results on the PET tracers for TSPO which could be explained by the different binding properties of the various PET tracers but also by methodological issues when quantifying the PET signal, such as high signal to noise ratio and low specificity [13–15]. A second generation of TSPO radioligands, including [^18^F]DPA-713, [^18^F]DPA-714, [^11^C]PBR28 etc., have been developed with the aim to increase their specificity, compared to the first generation. However, it was observed that their binding capability is affected by a single nucleotide polymorphism (SNP) in the TSPO gene denoted rs6971, an Alanine–Threonine substitution at base 147 which causes low, mixed or high-affinity binding in patients [16]. The SNP became an issue in the interpretation of the findings, leading to the current development of a third generation of TSPO radioligands, such as [^18^F]GE-180. The cerebellum is often used as a pseudo-reference region to assess the cerebral TSPO PET radiotracer binding without the need for arterial blood sampling [4, 17]. Indeed, there is no true reference region as TSPO is expressed in all brain areas. To be used as a pseudo-reference region for basal binding measurements, the cerebellum requires its TSPO concentration to be low and consistent during the disease progression, to detect small binding increases in other regions. Interestingly, the neuropathological status of the cerebellum, in terms of pathology (Braak), microglia and TSPO expression, during the course of AD is currently unclear.

The aims of our study were to: (i) characterise Aβ and tau pathology in the temporal and cerebellar cortex; (ii) explore the expression of TSPO and other microglial markers through the course of the disease; (iii) compare TSPO expression and AD pathology between both regions, particularly in view of the use of the cerebellum as a reference region for in vivo TSPO PET scans; (iv) assess the inflammatory microenvironment of both regions; and (v) determine if the rs6971 polymorphism affects TSPO immunoexpression.

## Materials and methods

### Cases

Brain tissue from 60 donors was sourced from the South–West Dementia Brain Bank and matched as closely as possible for age, sex and *post-mortem* delay between groups (Table 1). Cases were selected based on the Braak stage to allow exploration of the development of the pathology and TSPO expression. Formalin-fixed paraffin embedded tissue from the middle/superior temporal gyrus and cerebellum was obtained for immunohistological analysis and frozen tissue from the same area and same cases were used to assess the inflammatory environment with the Mesoscale Discovery (MSD) multiplex assay.

**Table 1:** Characteristics of the cases

Cases	Braak stages 0–II	Braak stages III–IV	Braak stages V–VI
Sex	7M:13F	11M:9F	9M:11F
Age at death (years, mean ± SD)	84.95 ± 8.9	86.20 ± 6.4	80.45 ± 7.6
Braak stage	0 = 4 I = 8 II = 8	III = 10 IV = 10	V = 8 VI = 12
APOE genotype	2/2 = 0 2/3 = 3 2/4 = 0 3/3 = 12 3/4 = 3 4/4 = 0 n/a = 2	2/2 = 0 2/3 = 4 2/4 = 0 3/3 = 8 3/4 = 8 4/4 = 0	2/2 = 0 2/3 = 1 2/4 = 1 3/3 = 10 3/4 = 5 4/4 = 3
Post-mortem delay (hours, mean ± SD)	54.00 ± 32.00	42.56 ± 19.59	37.00 ± 22.03
Total	20	20	20

*M* male, *F* female, *SD* standard deviation, *APOE* genotype, *n/a* not available

### Immunohistochemistry

6 μm sections of formalin-fixed paraffin-embedded tissue were used to perform immunohistochemistry to target: pan-Aβ (clone 4G8, Biolegend), phosphorylated tau (pTau, clone AT8, ThermoScientific MN1020) and TSPO (rabbit monoclonal anti-PBR antibody targeting TSPO, Abcam 109497) (Table 2). Microglial antibodies employed were: Iba1 (rabbit polyclonal, Wako labs 019-19741), HLA–DR (clone CR3/43, Dako M0775) and macrophage scavenger receptor (MSR)-A (polyclonal goat, R&D AF2708) (Table 2). Antibodies were visualised using the appropriate biotinylated secondary antibodies and the avidin–biotin-peroxidase complex method (Vectastain Elite, Vector Laboratories) with 3,3′-diaminobenzidine (DAB) as the chromogen and 0.05% hydrogen peroxide as the substrate (Vector Laboratories). The sections were counterstained with haematoxylin, dehydrated and mounted with Pertex (Histolab Products AB). A negative control with no primary antibody was included in all runs.

**Table 2:** Characteristics of the antibodies

Antibody	Species	Dilution	Supplier	Associated function/detection
Pan-Aβ (4G8)	Mouse	1:2000	Covance-Biolegend	Aβ pathology
pTau (AT8)	Mouse	1:500	Thermoscientific	Tau pathology
TSPO	Rabbit	1:5000	Abcam	Microglial mitochondria [10]
Iba1	Rabbit	1:750	Wako	Microglial motility and homeostasis [18]
HLA–DR	Mouse	1:200	Dako	Antigen presentation [7]
MSR-A	Goat	1:500	R&D	Microglial scavenging receptor with high affinity for Aβ [7]

### Image acquisition and analysis

Scanned images of the staining were obtained with the Olympus VS110 automated slide scanner (Olympus America Inc.) at 20× magnification. For each slide, 30 regions of interest (ROIs) of 500 × 500 µm were extracted in the same anatomical region of grey matter using the CSG add-on function to the Olympus VS-Desktop software [6]. ROIs were analysed with Fiji ImageJ v1.53c software (NIH, USA) [19] using an automated macro. For each antibody, a threshold was selected which included only specific staining and this threshold was then applied to all analyses with that antibody. The area fraction labelled by the antibody in each ROI was obtained by quantifying the presence or absence of the staining in each pixel and expressed as protein load (%). Protein loads were obtained by calculating the mean of the 30 images for each area of each case.

### Rs6971 genotyping

Cerebellar samples were genotyped for the SNP rs6971 using the PureLink™ Genomic DNA Extraction Mini Kit (ThermoFisher, K182001) to extract DNA as per manufacturers protocol. Purified genomic DNA concentration was established using the NanoDrop™ ND-1000 Microvolume Spectrometer and diluted to a final concentration of 0.9ng/µl in DNAse free water. The TaqMan^®^ SNP Genotyping assay kit (ThermoFisher, C_2512465_20), which contained forward/reverse primers and fluorescent VIC/FAM probes to correspond to A/G DNA bases, was used along with the 2X TaqMan^®^ Genotyping Master Mix (ThermoFisher, 4371353). The following cycle program was performed on the Applied Biosystems StepOnePlus™ Real-Time PCR system: 95 °C for 10min (HOLD) then 40 cycles of 95 °C × 15secs, 60 °C × 1min.

### Inflammatory environment assay

Inflammatory proteins were measured using the V-Plex Mesoscale Discovery (MSD) multi-spot assay platform (MesoScale Diagnostics, Rockville USA). Frozen samples of grey matter were prepared using a lysis solution made of: RIPA buffer (ThermoFisher, 89900), protease inhibitors (Sigma, 04693124001) and phosphatase inhibitors (ThermoFisher, 88667) for manual homogenisation via a Hybaid Ribolyser (Bio-Rad, #3589158). Brain homogenates were used for the V-Plex Chemokine Panel 1 (Eotaxin, Eotaxin-2, TARC, IP10, MIP1α, MIP1β, IL8, MCP1, MDC, and MCP4), Cytokine Panel 1 (GM-CSF, IL1α, IL5, IL7, IL12/IL23p40, IL15, IL16, IL17A, TNFβ, VEGF) and Proinflammatory Panel 1 (IFNγ, IL1β, IL2, IL4, IL6, IL8, IL10, IL12p70, IL13, TNFα). Each plate was read on a Meso Quickplex SQ120 with absolute target protein levels (pg/ml) obtained and normalised to the total protein amount [calculated via BCA assay (ThermoFisher, 23225)].

### Statistical analysis

Statistical analysis was carried out using the IBM SPSS v28 statistical software package (SPSS Inc. Chicago IL) and GraphPad Prism v9.2 (GraphPad Software. San Diego CA) for the graphs. For each marker, normality of the distribution was assessed by the Shapiro–Wilk test, and the distribution was observed to be non-parametric for all markers except TSPO in the temporal lobe. Comparisons between the different Braak stage groups were carried out using the non-parametric Kruskal–Wallis test followed by Dunn’s post-hoc test if significant, and the parametric one-way ANOVA test with the Tukey’s post-hoc test for TSPO in the temporal lobe. Comparisons between the temporal lobe and cerebellum were performed using Mann–Whitney *U* test for all markers. Correlations between the different markers were performed using either the Spearman’s test or the Pearson’s test depending on normality. To account for multiple correlation testing, the two-stage step-up Benjamini, Kreiger and Yekutieli test was used to control for the false discovery rate (FDR) in *post-hoc* analysis. Of note, correlation analysis was performed between the *post-mortem* tissue and all immunomarkers to ensure that the *post-mortem* delay did not influence the staining (Additional file 1: Table S5). Adjusted *P* values less than 0.05 for intergroup comparisons and 0.01 for correlations were considered significant.

## Results

### *A*β* and tau immunohistochemistry*

**Fig. 1:**
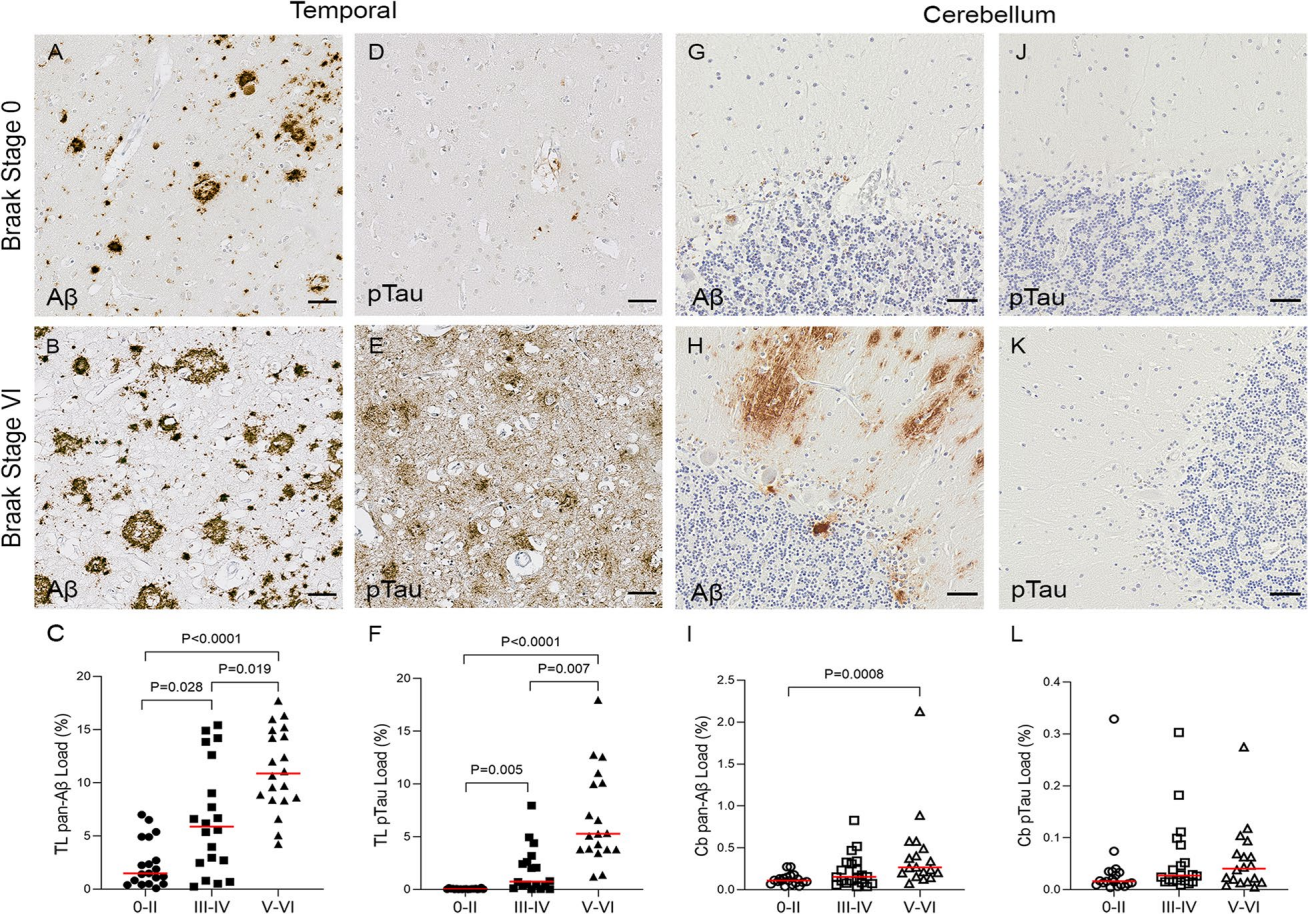
Illustrations and quantification of the immunostaining of Aβ (4G8) and pTau (AT8) expressed as protein load (%) in the temporal cortex (TL: **A**–**F**) and cerebellar cortex (Cb: **G**–**L**). Significant increases over the course of the disease are observed for Aβ (*P* < 0.0001) and pTau (*P* < 0.0001) in the temporal cortex and for Aβ (*P* = 0.0008) in the cerebellar cortex. No change was detected for pTau in the cerebellum. Counterstaining: Haematoxylin. Scale bar = 50μm

Aβ and pTau immunostaining was quantified in the temporal and cerebellar cortex to explore the differences in severity of AD pathology within these two brain areas. In the temporal cortex, at Braak stages 0–II, plaques were predominantly diffuse in morphology, with dense-cored plaques appearing at later Braak stages (Fig. 1A, B). Quantification showed a significant progressive increase in Aβ load through the Braak stages (Braak 0–II median 1.48%; Braak III–IV median 5.88%; Braak V–VI median 10.87%, *P* < 0.0001) (Fig. 1C). At Braak stages 0–II, very little pTau was present and mainly observed in neuronal cell bodies, whereas at Braak stages V–VI extensive pTau spread had occurred with the presence of neuropil threads and dystrophic neurites (Fig. 1D, E). Quantification showed a significant progressive increase in pTau load through the Braak stages (Braak 0–II median 0.04%; Braak III–IV median 0.75%; Braak V–VI median 5.27%, *P* < 0.0001) (Fig. 1F).

In the cerebellar cortex, Aβ deposition was mainly diffuse in pattern, without dense cores, and primarily distributed in the molecular layer (Fig. 1G, H). There was a significant increase in Aβ load across the Braak stages in the cerebellum (Braak 0–II median 0.11%; Braak III–IV median 0.15%; Braak V–VI median 0.27%, *P* = 0.0008) (Fig. 1I). pTau was not altered in the cerebellar cortex (*P* = 0.081) (Fig. 1J–L).

**Fig. 2:**
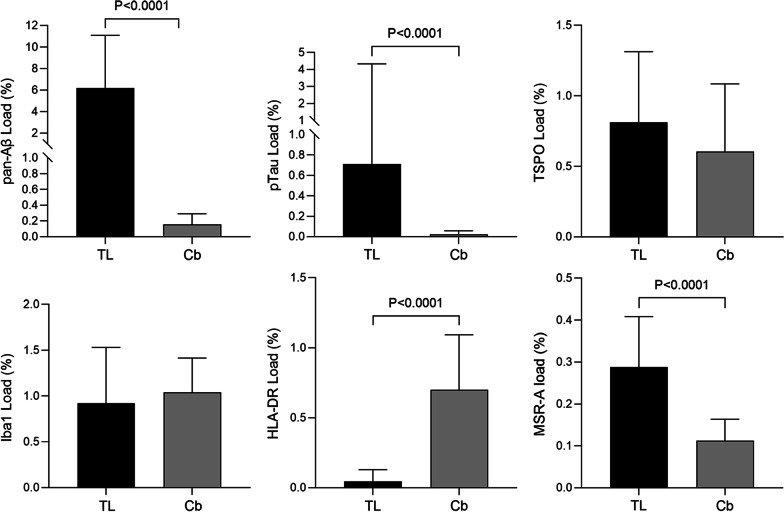
Comparisons between temporal lobe (TL) and cerebellum (Cb) for Aβ, pTau, TSPO and other microglial markers. Significant difference in load between TL and Cb for Aβ (*P* < 0.0001), pTau (*P* < 0.0001), HLA–DR (*P* < 0.0001) and MSR-A (*P* < 0.0001). No difference found for TSPO (*P* = 0.072) or Iba1 (*P* = 0.537)

Comparing the regions, both Aβ and pTau loads were significantly lower in the cerebellum than in the temporal cortex when examining all cases (Aβ: Cb median 0.16%; TL median 6.19%; pTau: Cb median 0.02%; TL median 0.71%, *P* < 0.0001) (Fig. 2).

### TSPO immunohistochemistry

**Fig. 3:**
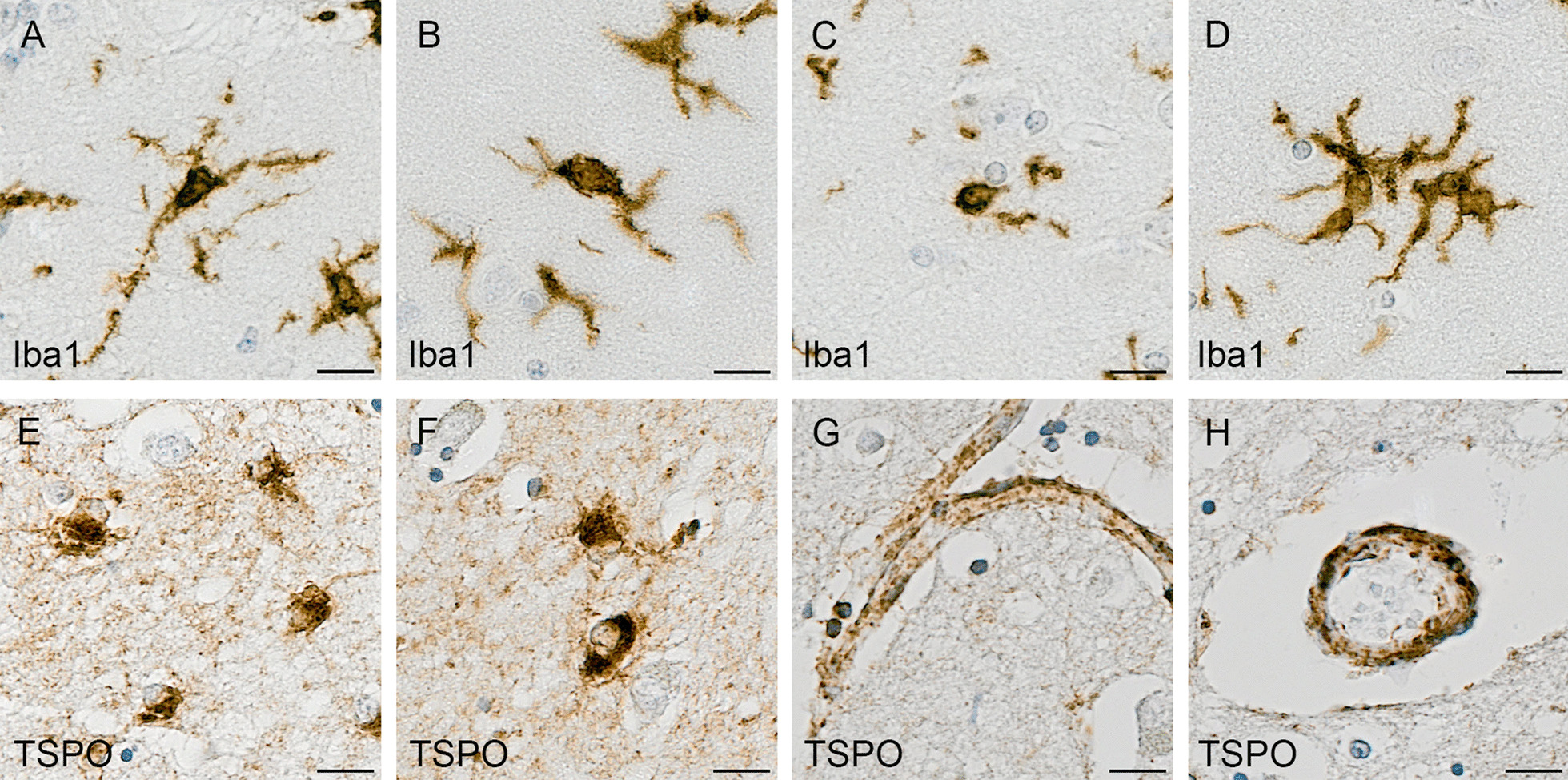
Illustrations of Iba1 and TSPO staining. Iba1 identifies: **A** ramified microglia, **B** intermediate microglial morphology with shorter processes, **C** amoeboid microglia, and **D** microglial cluster. **E**, **F** shows TSPO + microglia, with TSPO primarily surrounding the nuclei but staining also seen in some processes. **G**, **H** TSPO expression in the endothelial cells/smooth muscle cells of blood vessel walls, in the **G** longitudinal and **H** horizontal plane. Counterstaining: Haematoxylin. Scale bars = 50um

**Fig. 4:**
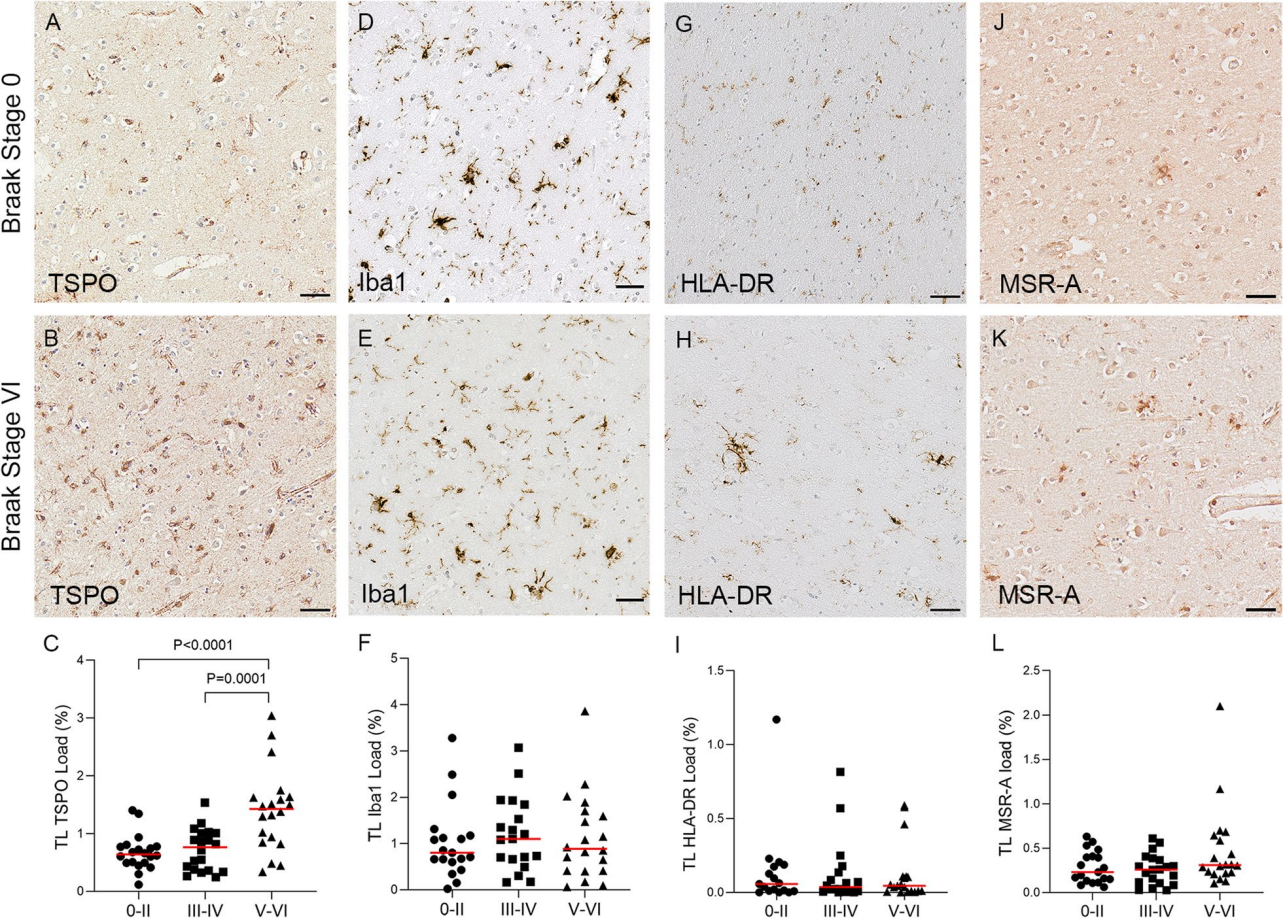
Illustrations and quantification in the temporal lobe (TL) of the immunolabelling expressed as protein load (%) for the microglial markers TSPO (**A**–**C**), Iba1 (**D**–**F**), HLA–DR (**G**–**I**) and MSR-A (**J**–**L**). A significant increase with Braak stage was seen for TSPO load (*P* < 0.0001), while no difference between Braak stages was detected for the other microglial markers. Counterstaining: Haematoxylin. Scale bars = 50μm

**Fig. 5:**
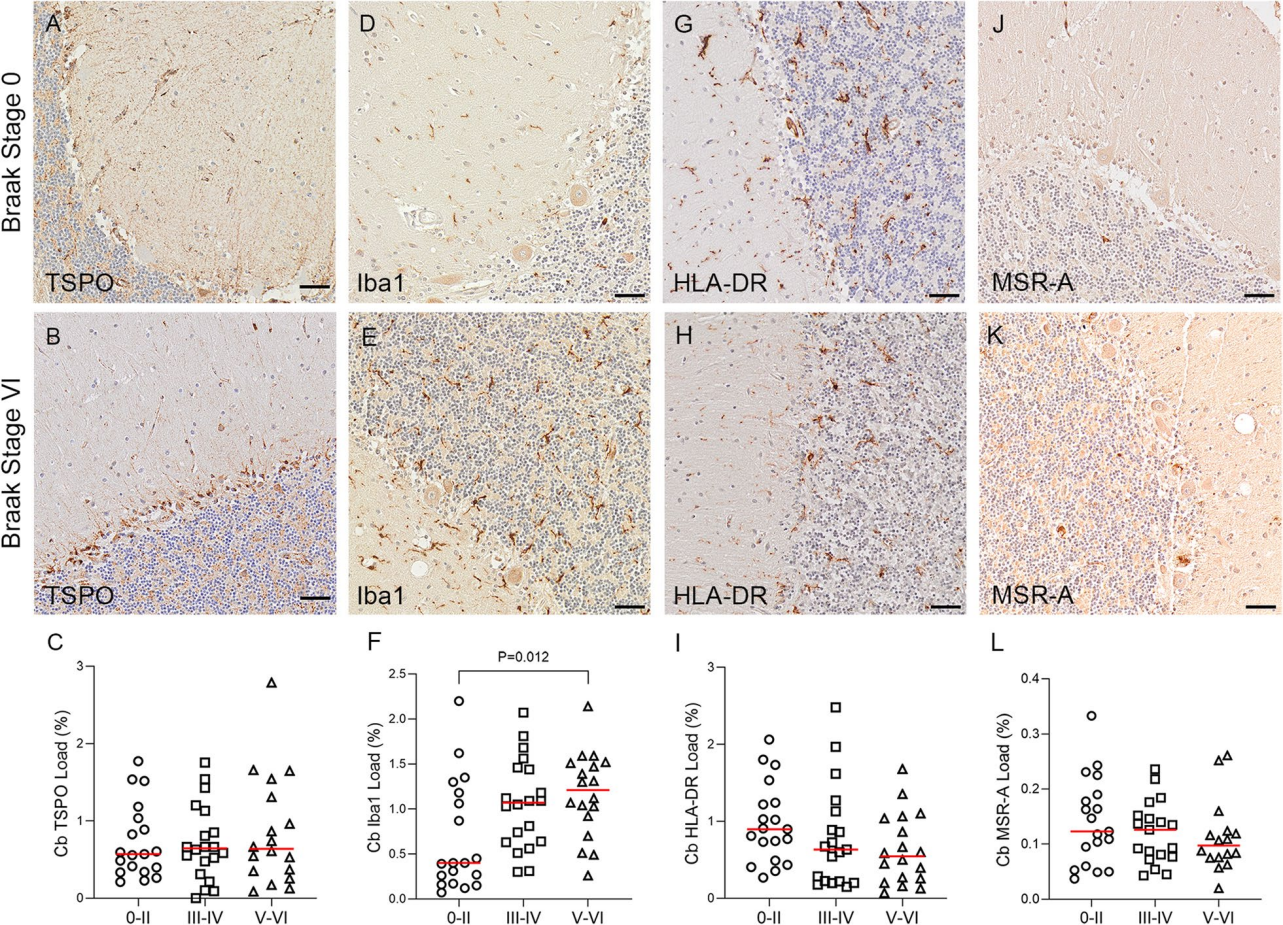
Illustrations and quantification in the cerebellar cortex (Cb) of immunostaining expressed as protein load (%) for the microglial markers TSPO (**A**–**C**), Iba1 (**D**–**F**), HLA–DR (**G**–**I**) and MSR-A (**J**–**L**). A significant increase with Braak stage was seen for Iba1 load (*P* = 0.012). Counterstaining: Haematoxylin. Scale bars = 50μm

TSPO immunostaining showed an intracellular dot-like pattern, consistent with labelling of mitochondria, localised predominantly to microglia and endothelial cells (Fig. 3). TSPO staining was mostly concentrated around the nuclei with some staining apparent in microglial processes (Fig. 3E, F). Neurons and other glial cells were unlabelled, suggesting that TSPO is restricted to cells of mesodermal origin (i.e., microglia [20] and endothelial cells [21]) and not present in the mitochondria of cells derived from neuroectoderm. In the temporal cortex, the TSPO load was significantly increased at Braak stage V–VI compared to Braak stages 0–II or III–IV (Braak 0–II mean 0.67%; Braak III–IV mean 0.72%; Braak V–VI mean 1.41%, *P* < 0.0001 and *P* = 0.0001, respectively) (Fig. 4C). In the cerebellum, TSPO exhibited a consistent pattern of staining particularly at the junction of the molecular and granular grey matter layers in Braak stage VI (Fig. 5B). However, there was no overall difference in TSPO load with Braak stage in this region (Braak 0–II median 0.57%; Braak III–IV median 0.65%; Braak V–VI median 0.64%, *P* = 0.925) (Fig. 5C).

### Other microglial proteins

In the temporal lobe, Iba1 + and HLA–DR + microglia were predominantly ramified in morphology, whereas MSR-A + microglia appeared more amoeboid (Fig. 4). Clustering of microglia was observed with Iba1 (Fig. 3D), HLA–DR and MSR-A (Fig. 4H, k), mainly in Braak stage V–VI cases and consistent with localisation to Aβ plaques, as previously described [22]. However, unlike TSPO, quantification in the temporal cortex showed no significant change in load with Braak stage for Iba1 (Braak 0–II median 0.8%; Braak III–IV median 1.1%; Braak V–VI median 0.89%, *P* = 0.688), HLA–DR (Braak 0–II median 0.06%; Braak III–IV median 0.04%; Braak V–VI median 0.05%, *P* = 0.968) or MSR-A (Braak 0–II median 0.23%; Braak III–IV median 0.26%; Braak V–VI median 0.31%, *P* = 0.126) (Fig. 4F, I, L). In the cerebellar cortex, Iba1 + and HLA–DR + microglia were more ramified compared to those labelled with MSR-A which had an amoeboid shape (Fig. 5). In contrast to the temporal cortex, cerebellar Iba1 load progressively increased with Braak stage (Braak 0–II median 0.4%; Braak III–IV median 1.07%; Braak V–VI median 1.21%, *P* = 0.012) (Fig. 5F), whereas TSPO (Braak 0–II median 0.57%; Braak III–IV median 0.65%; Braak V–VI median 0.64%, *P* = 0.925), HLA–DR (Braak 0–II median 0.9%; Braak III–IV median 0.63%; Braak V–VI median 0.54%, *P* = 0.1) and MSR-A (Braak 0–II median 0.12%; Braak III–IV median 0.13%; Braak V–VI median 0.1%, *P* = 0.531) loads were not changed (Fig. 5C, I, L).

Comparison between the temporal and cerebellar cortices did not reveal differences in Iba1 (TL median 0.92%; Cb median 1.04%, *P* = 0.537) or TSPO (TL median 0.81%; Cb median 0.61%, *P* = 0.072) loads, irrespective of Braak stage. However, there were significantly less HLA–DR in the temporal lobe than the cerebellum (TL median 0.046%; Cb median 0.7%, *P* < 0.0001) and vice versa for MSR-A (TL median 0.288%; Cb median 0.113%, *P* < 0.0001) (Fig. 2).

### TSPO Rs6971 genotyping

**Fig. 6:**
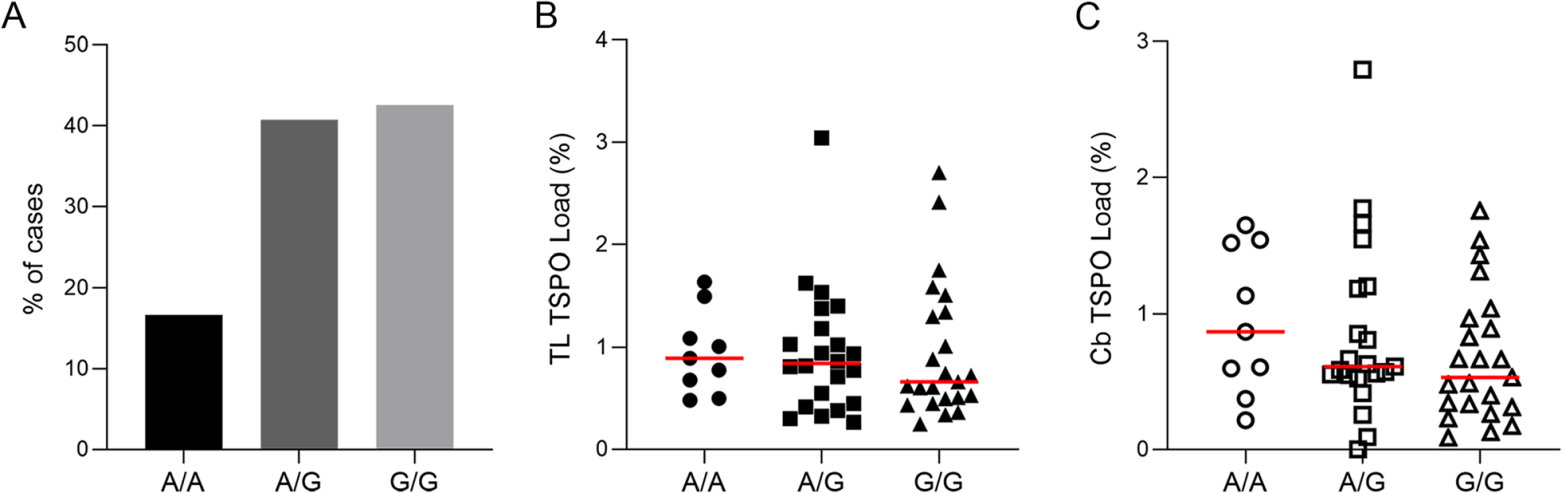
rs6971 genotyping of the cohort with **A** percentage of cases for each genotype defined as: A/A (low-affinity binder), A/G (mixed-affinity binder) or G/G(high-affinity binder)). **B**, **C** comparisons between each genotype and TSPO protein load (%) in the temporal lobe (TL) and cerebellum (Cb), respectively, no significant change detected

The genotyping results revealed that 9/54 (16.67%) of cases were homozygous for A/A (low-affinity binding for TSPO PET ligand), 22/54 (40.74%) were heterozygous for A/G (mixed-affinity binding) and 23/54 (42.59%) were homozygous for G/G (high-affinity binding) (Fig. 6A), consistent with other population data [23]. There was no significant differences between genotypes when comparing TSPO protein load in the temporal lobe (A/A median 0.89%; A/G median 0.84%; G/G median 0.66%, *P* = 0.77) or the cerebellum (A/A median 0.87%; A/G median 0.61%; G/G median 0.53%, *P* = 0.37) (Fig. 6B, C). This shows that the SNP does not affect the TSPO immunostaining.

### Correlations

Correlations were performed to assess whether Aβ, pTau, TSPO and other microglial markers were related amongst each other and between the temporal lobe and cerebellum. Across all Braak stages, temporal Aβ was positively correlated with cerebellar Aβ (*r*_s_ = 0.505, *P* < 0.001), and temporal pTau with both temporal Aβ (*r*_s_ = 0.751, *P* < 0.001) and cerebellar Aβ (*r*_s_ = 0.516, *P* < 0.001). Significant positive correlations were also observed for temporal TSPO with temporal pTau (*r*_s_ = 0.465, *P* < 0.001) and temporal MSR-A (*r*_s_ = 0.356, *P* = 0.005) (Table 3).

**Table 3:** Correlations of pathological and microglial markers across the temporal (TL) and cerebellar (Cb) cortex (all Braak stages)

	Aβ (Cb)	pTau (TL)	pTau( Cb)	TSPO (TL)	TSPO (Cb)	Iba1 (TL)	Iba1 (Cb)	HLA–DR (TL)	HLA–DR (Cb)	MSR-A (TL)	MSR-A (Cb)
Aβ (TL)	***r***_**s**_** = 0.505** ***P* < 0.001**	***r***_**s**_** = 0.751** ***P* < 0.001**	*r*_s_ = 0.287 *P* = 0.032	*r*_s_ = 0.268 *P* = 0.04	*r*_s_ = 0.176 *P* = 0.193	*r*_s_ = 0.019 *P* = 0.889	*r*_s_ = 0.295 *P* = 0.027	*r*_s_ = 0.042 *P* = 0.765	*r*_s_ = *− *0.118 *P* = 0.392	*r*_s_ = 0.061 *P* = 0.646	*r*_s_ = *− *0.033 *P* = 0.812
Aβ (Cb)		***r***_**s**_** = 0.516** ***P* < 0.001**	*r*_s_ = 0.177 *P* = 0.187	*r*_s_ = 0.296 *P* = 0.026	*r*_s_ = 0.272 *P* = 0.04	*r*_s_ = 0.133 *P* = 0.33	*r*_s_ = 0.219 *P* = 0.102	*r*_s_ = 0.065 *P* = 0.647	*r*_s_ = *− *0.143 *P* = 0.294	*r*_s_ = 0.121 *P* = 0.37	*r*_s_ = *− *0.013 *P* = 0.927
pTau (TL)			*r*_s_ = 0.339 *P* = 0.012	***r***_**s**_** = 0.465** ***P* < 0.001**	*r*_s_ = 0.095 *P* = 0.494	*r*_s_ = 0.100 *P* = 0.463	*r*_s_ = 0.319 *P* = 0.019	*r*_s_ = 0.129 *P* = 0.368	*r*_s_ = *− *0.23 *P* = 0.098	*r*_s_ = 0.099 *P* = 0.465	*r*_s_ = *− *0.184 *P* = 0.191
pTau (Cb)				*r*_s_ = 0.081 *P* = 0.547	*r*_s_ = 0.141 *P* = 0.297	*r*_s_ = 0.079 *P* = 0.561	*r*_s_ = *− *0.004 *P* = 0.979	*r*_s_ = *− *0.036 *P* = 0.802	*r*_s_ = *− *0.084 *P* = 0.539	*r*_s_ = 0.104 *P* = 0.442	*r*_s_ = 0.214 *P* = 0.12
TSPO (TL)					*r*_s_ = 0.220 0 = 0.101	*r*_s_ = 0.010 *P* = 941	*r*_s_ = 0.284 *P* = 0.033	*r*_s_ = 0.139 *P* = 0.316	*r*_s_ = 0.083 *P* = 0.544	***r***_**s**_** = 0.356** ***P* = 0.005**	*r*_s_ = *− *0.071 *P* = 0.612
TSPO (Cb)						*r*_s_ = *− *0.091 *P* = 0.507	*r*_s_ = 0.230 *P* = 0.085	*r*_s_ = 0.274 *P* = 0.05	*r*_s_ = 0.164 *P* = 0.228	*r*_s_ = 0.204 *P* = 0.129	*r*_s_ = 0.015 *P* = 0.913
Iba1 (TL)							*r*_s_ = 0.256 *P* = 0.057	*r*_s_ = 0.082 *P* = 0.56	*r*_s_ = *− *0.008 *P* = 0.955	*r*_s_ = *− *0.103 *P* = 0.436	*r*_s_ = *− *0.279 *P* = 0.043
Iba1 (Cb)								*r*_s_ = 0.119 *P* = 0.402	*r*_s_ = *− *0.011 *P* = 0.933	*r*_s_ = 0.077 *P* = 0.569	*r*_s_ = *− *0.071 *P* = 0.61
HLA–DR (TL)									***r***_**s**_** = 0.548** ***P* < 0.001**	*r*_s_ = 0.244 *P* = 0.075	*r*_s_ = *− *0.08 *P* = 0.583
HLA–DR (Cb)										*r*_s_ = 0.088 *P* = 0.518	*r*_s_ = 0.087 *P* = 0.536
MSR-A (TL)											*r*_s_ = 0.043 *P* = 0.759

*r*_*s*_ Spearman’s rank correlation, significant *P* values are in bold

*TL* temporal lobe; *Cb* cerebellum

### Neuroinflammatory environment

To characterise the neuroinflammatory environment, 30 inflammatory analytes were assessed. Only the cytokine IL15 increased progressively with Braak stage in the temporal cortex (*P* = 0.0189) (Additional file 1: Table S2). No other significant changes were identified with Braak stage in either the temporal cortex or cerebellum (Additional file 1: Tables S1 and S3).

In terms of the relationships between Aβ, pTau, TSPO, other microglial proteins and the inflammatory molecules in the temporal cortex, there were significant negative correlations between TSPO and GM-CSF (*r*_s_ = − 0.355, *P* < 0.01) and between HLA–DR and macrophage-derived cytokine (MDC) (*r*_s_ = − 0.381, *P* = 0.007) (Table 4). In the cerebellum, there was a significant positive correlation between Iba1 and IL16 (*r*_s_ = 0.438, *P* < 0.001) (Table 5). Directly comparing the protein concentration in both regions, negating Braak stage, most markers were significantly increased in the temporal lobe compared to the cerebellum (Additional file 1: Table S4). However, several of the inflammatory proteins were increased in the cerebellum, including VEGF, IL8HA, IL10 and IL2 (Additional file 1: Table S4).

**Table 4:** Correlations between pathological/microglial proteins and inflammatory markers across all Braak stages in the temporal lobe

	Aβ	pTau	TSPO	Iba1	HLA–DR	MSR-A
GM-CSF	*r*_s_ = − 0.145 *P* = 0.312	*r*_s_ = − 0.230 *P* = 0.111	***r***_**s**_** = − 0.355** ***P* < 0.01**	*r*_s_ = − 0.154 *P* = 0.280	*r*_s_ = − 0.088 *P* = 0.554	*r*_s_ = − 0.023 *P* = 0.875
IL1α	*r*_s_ = 0.098 *P* = 0.0499	*r*_s_ = − 0.025 *P* = 0.863	*r*_s_ = − 0.136 *P* = 0.337	*r*_s_ = − 0.103 *P* = 0	*r*_s_ = 0.017 *P* = 0.910	*r*_s_ = − 0.065 *P* = 0.652
IL12/IL23p70	*r*_s_ = − 0.104 *P* = 0.472	*r*_s_ = − 0.192 *P* = 0.187	*r*_s_ = − 0.105 *P* = 0.458	*r*_s_ = 0.060 *P* = 0.676	*r*_s_ = 0.201 *P* = 0.176	*r*_s_ = − 0.084 *P* = 0.560
IL15	*r*_s_ = 0.123 *P* = 0.393	*r*_s_ = 0.268 *P* = 0.063	*r*_s_ = 0.310 *P* = 0.025	*r*_s_ = − 0.094 *P* = 0.513	*r*_s_ = − 0.005 *P* = 0.971	*r*_s_ = 0.044 *P* = 0.757
IL16	*r*_s_ = 0.039 *P* = 0.786	*r*_s_ = 0.060 *P* = 0.682	*r*_s_ = 0.155 *P* = 0.274	*r*_s_ = 0.267 *P* = 0.059	*r*_s_ = 0.202 *P* = 0.174	*r*_s_ = − 0.161 *P* = 0.260
IL17A	*r*_s_ = 0.016 *P* = 0.915	*r*_s_ = 0.206 *P* = 0.156	*r*_s_ = 0.134 *P* = 0.344	*r*_s_ = − 0.145 *P* = 0.310	*r*_s_ = − 0.239 *P* = 0.106	*r*_s_ = − 0.154 *P* = 0.280
IL5	*r*_s_ = − 0.117 *P* = 0.417	*r*_s_ = − 0.053 *P* = 0.718	*r*_s_ = − 0.107 *P* = 0.451	*r*_s_ = 0.091 *P* = 0.523	*r*_s_ = 0.160 *P* = 0.283	*r*_s_ = − 0.009 *P* = 0.947
IL7	*r*_s_ = − 0.297 *P* = 0.036	*r*_s_ = − 0.285 *P* = 0.047	*r*_s_ = − 0.013 *P* = 0.925	*r*_s_ = − 0.123 *P* = 0.390	*r*_s_ = 0.144 *P* = 0.334	*r*_s_ = 0.004 *P* = 0.979
TNFβ	*r*_s_ = − 0.069 *P* = 0.632	*r*_s_ = − 0.175 *P* = 0.229	*r*_s_ = 0.042 *P* = 0.767	*r*_s_ = − 0.065 *P* = 0.649	*r*_s_ = − 0.007 *P* = 0.962	*r*_s_ = − 0.021 *P* = 0.883
VEGF	*r*_s_ = − 0.310 *P* = 0.028	*r*_s_ = − 0.081 *P* = 0.578	*r*_s_ = − 0.188 *P* = 0.182	*r*_s_ = 0.010 *P* = 0.945	*r*_s_ = 0.170 *P* = 0.254	*r*_s_ = 0.152 *P* = 0.286
Eotaxin	*r*_s_ = 0.005 *P* = 0.974	*r*_s_ = 0.207 *P* = 0.153	*r*_s_ = 0.019 *P* = 0.895	*r*_s_ = 0.063 *P* = 0.662	*r*_s_ = − 0.256 *P* = 0.083	*r*_s_ = − 0.092 *P* = 0.520
Eotaxin 3	*r*_s_ = 0.0003 *P* = 0.998	*r*_s_ = 0.110 *P* = 0.451	*r*_s_ = − 0.014 *P* = 0.923	*r*_s_ = − 0.106 *P* = 0.461	*r*_s_ = − 0.209 *P* = 0.158	*r*_s_ = 0.001 *P* = 0.993
IL8 (HA)	*r*_s_ = − 0.176 *P* = 0.220	*r*_s_ = 0.092 *P* = 0.531	*r*_s_ = 0.067 *P* = 0.639	*r*_s_ = 0.016 *P* = 0.914	*r*_s_ = − 0.156 *P* = 0.296	*r*_s_ = 0.036 *P* = 0.800
IP10	*r*_s_ = 0.051 *P* = 0.725	*r*_s_ = 0.192 *P* = 0.186	*r*_s_ = 0.149 *P* = 0.293	*r*_s_ = 0.251 *P* = 0.075	*r*_s_ = 0.018 *P* = 0.906	*r*_s_ = 0.059 *P* = 0.682
MCP1	*r*_s_ = 0.186 *P* = 0.196	*r*_s_ = 0.342 *P* = 0.016	*r*_s_ = 0.119 *P* = 0.400	*r*_s_ = 0.072 *P* = 0.614	*r*_s_ = − 0.054 *P* = 0.718	*r*_s_ = − 0.003 *P* = 0.986
MCP4	*r*_s_ = − 0.0004 *P* = 0.998	*r*_s_ = 0.250 *P* = 0.083	*r*_s_ = 0.335 *P* = 0.015	*r*_s_ = − 0.084 *P* = 0.557	*r*_s_ = − 0.219 *P* = 0.139	*r*_s_ = − 0.081 *P* = 0.574
MDC	*r*_s_ = 0.011 *P* = 0.942	*r*_s_ = 0.250 *P* = 0.084	*r*_s_ = 0.152 *P* = 0.281	*r*_s_ = − 0.070 *P* = 0.624	***r***_**s**_** = − 0.381** ***P* = 0.007**	*r*_s_ = − 0.003 *P* = 0.982
MIP1α	*r*_s_ = 0.002 *P* = 0.991	*r*_s_ = 0.250 *P* = 0.084	*r*_s_ = 0.054 *P* = 0.702	*r*_s_ = 0.060 *P* = 0.675	*r*_s_ = − 0.150 *P* = 0.315	*r*_s_ = 0.002 *P* = 0.990
MIP1β	*r*_s_ = − 0.188 *P* = 0.192	*r*_s_ = 0.103 *P* = 0.479	*r*_s_ = − 0.010 *P* = 0.944	*r*_s_ = 0.021 *P* = 0.883	*r*_s_ = − 0.140 *P* = 0.350	*r*_s_ = − 0.026 *P* = 0.854
TARC	*r*_s_ = − 0.015 *P* = 0.919	*r*_s_ = 0.046 *P* = 0754	*r*_s_ = − 0.177 *P* = 0.209	*r*_s_ = 0.0005 *P* = 0.970	*r*_s_ = − 0.075 *P* = 0.618	*r*_s_ = 0.008 *P* = 0.957
IFNγ	*r*_s_ = − 0.030 *P* = 0.835	*r*_s_ = − 0.005 *P* = 0.971	*r*_s_ = − 0.123 *P* = 0.386	*r*_s_ = 0.032 *P* = 0.821	*r*_s_ = 0.019 *P* = 0.897	*r*_s_ = 0.141 *P* = 0.325
IL1β	*r*_s_ = − 0.121 *P* = 0.401	*r*_s_ = 0.039 *P* = 0.791	*r*_s_ = − 0.088 *P* = 0.537	*r*_s_ = 0.088 *P* = 0.537	*r*_s_ = − 0.005 *P* = 0.974	*r*_s_ = − 0.025 *P* = 0.864
IL10	*r*_s_ = − 0.086 *P* = 0.550	*r*_s_ = 0.049 *P* = 0.741	*r*_s_ = − 0.168 *P* = 0.233	*r*_s_ = − 0.023 *P* = 0.873	*r*_s_ = − 0.128 *P* = 0.391	*r*_s_ = − 0.058 *P* = 0.684
IL12p70	*r*_s_ = − 0.163 *P* = 0.258	*r*_s_ = − 0.049 *P* = 0.739	*r*_s_ = 0.027 *P* = 0.850	*r*_s_ = − 0.236 *P* = 0.095	*r*_s_ = − 0.169 *P* = 0.255	*r*_s_ = 0.002 *P* = 0.990
IL13	*r*_s_ = − 0.169 *P* = 0.241	*r*_s_ = − 0.016 *P* = 0.914	*r*_s_ = 0.000 *P* = 0.999	*r*_s_ = − 0.273 *P* = 0.053	*r*_s_ = − 0.255 *P* = 0.084	*r*_s_ = 0.155 *P* = 0.276
IL2	*r*_s_ = − 0.142 *P* = 0.324	*r*_s_ = − 0.095 *P* = 0.515	*r*_s_ = − 0.134 *P* = 0.345	*r*_s_ = − 0.061 *P* = 0.669	*r*_s_ = − 0.122 *P* = 0.413	*r*_s_ = 0.126 *P* = 0.337
IL4	*r*_s_ = − 0.229 *P* = 0.110	*r*_s_ = 0.016 *P* = 0.912	*r*_s_ = 0.072 *P* = 0.614	*r*_s_ = − 0.125 *P* = 0.382	*r*_s_ = − 0.111 *P* = 0.458	*r*_s_ = 0.159 *P* = 0.266
IL6	*r*_s_ = − 0.008 *P* = 0.958	*r*_s_ = 0.033 *P* = 0.820	*r*_s_ = 0.063 *P* = 0.655	*r*_s_ = 0.032 *P* = 0.823	*r*_s_ = − 0.057 *P* = 0.704	*r*_s_ = 0.115 *P* = 0.421
IL8	*r*_s_ = − 0.121 *P* = 0.401	*r*_s_ = 0.008 *P* = 0.954	*r*_s_ = − 0.031 *P* = 0.827	*r*_s_ = 0.000 P = 0.998	*r*_s_ = − 0.322 *P* = 0.027	*r*_s_ = − 0.025 *P* = 0.860
TNFα	*r*_s_ = − 0.273 *P* = 0.055	*r*_s_ = − 0.103 *P* = 0.480	*r*_s_ = − 0.118 *P* = 0.406	*r*_s_ = − 0.054 *P* = 0.708	*r*_s_ = − 0.221 *P* = 0.135	*r*_s_ = − 0.021 *P* = 0.883

*r*_s_ Spearman’s rank correlation, significant *P* values in bold

**Table 5:** Correlations between the pathological/microglial proteins and inflammatory markers across all Braak stages in the cerebellum

	Aβ	pTau	TSPO	Iba1	HLA–DR	MSR-A
GM-CSF	*r*_s_ = 0.114 *P* = 0.556	*r*_s_ = − 0.163 *P* = 0.398	*r*_s_ = − 0.237 *P* = 0.196	*r*_s_ = 0.031 *P* = 0.873	*r*_s_ = 0.049 *P* = 0.802	*r*_s_ = 0.136 *P* = 0.498
IL1α	*r*_s_ = 0.060 *P* = 0.674	*r*_s_ = 0.014 *P* = 0.920	*r*_s_ = − 0.121 *P* = 0.392	*r*_s_ = 0.086 *P* = 0.546	*r*_s_ = − 0.189 *P* = 0.179	*r*_s_ = 0.037 *P* = 0.800
IL12/IL23p70	*r*_s_ = 0.006 *P* = 0.969	*r*_s_ = − 0.074 *P* = 0.622	*r*_s_ = − 0.037 *P* = 0.807	*r*_s_ = 0.333 *P* = 0.022	*r*_s_ = − 0.194 *P* = 0.191	*r*_s_ = − 0.069 *P* = 0.654
IL15	*r*_s_ = − 0.005 *P* = 0.974	*r*_s_ = 0.184 *P* = 0.193	*r*_s_ = 0.241 *P* = 0.086	*r*_s_ = 0.151 *P* = 0.286	*r*_s_ = − 0.118 *P* = 0.403	*r*_s_ = 0.079 *P* = 0.584
IL16	*r*_s_ = 0.189 *P* = 0.179	*r*_s_ = − 0.001 *P* = 0.992	*r*_s_ = 0.121 *P* = 0.391	***r***_**s**_** = 0.438** ***P* < 0.001**	*r*_s_ = 0.034 *P* = 0.810	*r*_s_ = − 0.018 *P* = 0.902
IL17A	*r*_s_ = − 0.180 *P* = 0.202	*r*_s_ = − 0.025 *P* = 0.863	*r*_s_ = 0.058 *P* = 0.682	*r*_s_ = 0.075 *P* = 0.599	*r*_s_ = − 0.239 *P* = 0.088	*r*_s_ = − 0.011 *P* = 0.941
IL5	*r*_s_ = 0.255 *P* = 0.094	*r*_s_ = 0.004 *P* = 0.982	*r*_s_ = − 0.364 *P* = 0.015	*r*_s_ = 0.053 *P* = 0.733	*r*_s_ = − 0.177 *P* = 0.249	*r*_s_ = 0.005 *P* = 0.976
IL7	*r*_s_ = − 0.223 *P* = 0.464	*r*_s_ = 0.083 *P* = 0.789	*r*_s_ = 0.586 *P* = 0.035	*r*_s_ = 0.270 *P* = 0.372	*r*_s_ = 0.193 *P* = 0.528	*r*_s_ = − 0.182 *P* = 0.571
TNFβ	*r*_s_ = − 0.296 *P* = 0.049	*r*_s_ = − 0.199 *P* = 0.189	*r*_s_ = − 0.088 *P* = 0.567	*r*_s_ = 0.172 *P* = 0.260	*r*_s_ = − 0.220 *P* = 0.146	*r*_s_ = − 0.059 *P* = 0.709
VEGF	*r*_s_ = − 0.063 *P* = 0.655	*r*_s_ = − 0.088 *P* = 0.537	*r*_s_ = − 0.206 *P* = 0.142	*r*_s_ = − 0.070 *P* = 0.622	*r*_s_ = − 0.229 *P* = 0.102	*r*_s_ = − 0.096 *P* = 0.507
Eotaxin	*r*_s_ = − 0.129 *P* = 0.361	*r*_s_ = − 0.206 *P* = 0.142	*r*_s_ = − 0.226 *P* = 0.108	*r*_s_ = 0.177 *P* = 0.209	*r*_s_ = − 0.212 *P* = 0.131	*r*_s_ = − 0.127 *P* = 0.379
Eotaxin 3	*r*_s_ = 0.058 *P* = 0.681	*r*_s_ = − 0.097 *P* = 0.493	*r*_s_ = − 0.077 *P* = 0.588	*r*_s_ = − 0.156 *P* = 0.269	*r*_s_ = 0.014 *P* = 0.922	*r*_s_ = 0.109 *P* = 0.449
IL8 (HA)	*r*_s_ = − 0.089 *P* = 0.532	*r*_s_ = 0.060 *P* = 0.673	*r*_s_ = − 0.023 *P* = 0.870	*r*_s_ = 0.056 *P* = 0.692	*r*_s_ = − 0.316 *P* = 0.023	*r*_s_ = 0.041 *P* = 0.775
I*P*10	*r*_s_ = − 0.059 *P* = 0.679	*r*_s_ = − 0.082 *P* = 0.563	*r*_s_ = 0.059 *P* = 0.680	*r*_s_ = − 0.036 *P* = 0.799	*r*_s_ = − 0.016 *P* = 0.911	*r*_s_ = 0.040 *P* = 0.783
MCP1	*r*_s_ = − 0.117 *P* = 0.410	*r*_s_ = − 0.043 *P* = 0.760	*r*_s_ = − 0.090 *P* = 0.524	*r*_s_ = 0.058 *P* = 0.658	*r*_s_ = − 0.151 *P* = 0.287	*r*_s_ = 0.129 *P* = 0.373
MCP4	*r*_s_ = − 0.153 *P* = 0.280	*r*_s_ = − 0.158 *P* = 0.264	*r*_s_ = − 0.166 *P* = 0.240	*r*_s_ = 0.148 *P* = 0.295	*r*_s_ = − 0.222 *P* = 0.114	*r*_s_ = − 0.099 *P* = 0.495
MDC	*r*_s_ = − 0.135 *P* = 0.341	*r*_s_ = − 0.015 *P* = 0.916	*r*_s_ = − 0.083 *P* = 0.557	*r*_s_ = 0.143 *P* = 0.311	*r*_s_ = − 0.233 *P* = 0.096	*r*_s_ = 0.049 *P* = 0.734
MIP1α	*r*_s_ = − 0.137 *P* = 0.332	*r*_s_ = − 0.144 *P* = 0.308	*r*_s_ = − 0.184 *P* = 0.191	*r*_s_ = 0.184 *P* = 0.191	*r*_s_ = − 0.253 *P* = 0.070	*r*_s_ = − 0.124 *P* = 0.389
MIP1β	*r*_s_ = − 0.299 *P* = 0.031	*r*_s_ = − 0.175 *P* = 0.215	*r*_s_ = − 0.193 *P* = 0.171	*r*_s_ = 0.119 *P* = 0.402	*r*_s_ = − 0.238 *P* = 0.090	*r*_s_ = 0.006 *P* = 0.969
TARC	*r*_s_ = − 0.303 *P* = 0.029	*r*_s_ = − 0.074 *P* = 0.603	*r*_s_ = − 0.169 *P* = 0.231	*r*_s_ = − 0.008 *P* = 0.957	*r*_s_ = − 0.142 *P* = 0.316	*r*_s_ = − 0.106 *P* = 0.463
IFNγ	*r*_s_ = 0.010 *P* = 0.946	*r*_s_ = − 0.227 *P* = 0.113	*r*_s_ = 0.164 *P* = 0.257	*r*_s_ = 0.336 *P* = 0.017	*r*_s_ = − 0.165 *P* = 0.251	*r*_s_ = − 0.150 *P* = 0.310
IL1β	*r*_s_ = − 0.099 *P* = 0.485	*r*_s_ = 0.028 *P* = 0.843	*r*_s_ = 0.070 *P* = 0.620	*r*_s_ = 0.011 *P* = 0.940	*r*_s_ = 0.058 *P* = 0.681	*r*_s_ = 0.178 *P* = 0.216
IL10	*r*_s_ = 0.014 *P* = 0.924	*r*_s_ = − 0.226 *P* = 0.107	*r*_s_ = − 0.061 *P* = 0.667	*r*_s_ = 0.321 *P* = 0.020	*r*_s_ = − 0.238 *P* = 0.089	*r*_s_ = 0.026 *P* = 0.858
IL12p70	*r*_s_ = − 0.042 *P* = 0.765	*r*_s_ = − 0.232 *P* = 0.098	*r*_s_ = − 0.111 *P* = 0.432	*r*_s_ = 0.287 *P* = 0.039	*r*_s_ = − 0.130 *P* = 0.360	*r*_s_ = − 0.019 *P* = 0.895
IL13	*r*_s_ = − 0.129 *P* = 0.361	*r*_s_ = − 0.146 *P* = 0.301	*r*_s_ = − 0.311 *P* = 0.025	*r*_s_ = − 0.137 *P* = 0.333	*r*_s_ = − 0.225 *P* = 0.108	*r*_s_ = − 0.010 *P* = 0.947
IL2	*r*_s_ = − 0.031 *P* = 0.829	*r*_s_ = − 0.242 *P* = 0.087	*r*_s_ = − 0.076 *P* = 0.596	*r*_s_ = 0.340 *P* = 0.015	*r*_s_ = − 0.244 *P* = 0.084	*r*_s_ = − 0.050 *P* = 0.734
IL4	*r*_s_ = 0.122 *P* = 0.387	*r*_s_ = − 0.030 *P* = 0.834	*r*_s_ = − 0.006 *P* = 0.968	*r*_s_ = 0.192 *P* = 0.174	*r*_s_ = − 0.196 *P* = 0.163	*r*_s_ = − 0.104 *P* = 0.473
IL6	*r*_s_ = − 0.063 *P* = 0.659	*r*_s_ = 0.032 *P* = 0.823	*r*_s_ = 0.048 *P* = 0.736	*r*_s_ = 0.055 *P* = 0.699	*r*_s_ = − 0.053 *P* = 0.711	*r*_s_ = 0.174 *P* = 0.228
IL8	*r*_s_ = 0.006 *P* = 0.967	*r*_s_ = 0.029 *P* = 0.836	*r*_s_ = − 0.092 *P* = 0.519	*r*_s_ = − 0.031 *P* = 0.829	*r*_s_ = − 0.098 *P* = 0.491	*r*_s_ = 0.159 *P* = 0.271
TNFα	*r*_s_ = − 0.143 *P* = 0.312	*r*_s_ = − 0.237 *P* = 0.091	*r*_s_ = − 0.200 *P* = 0.154	*r*_s_ = 0.276 *P* = 0.047	*r*_s_ = − 0.163 *P* = 0.247	*r*_s_ = − 0.052 *P* = 0.719

*r*_s_ Spearman’s rank correlation, significant *P* values in bold

## Discussion

In this study, we have explored the expression of the TSPO protein, a PET target used to image neuroinflammation, in the temporal lobe and cerebellum during the pathological course of AD, using Braak stages as markers of severity of the disease. Using a quantitative and automatic approach, Aβ and pTau deposition were investigated in both regions. Our unbiased assessment of key hallmarks of AD pathology demonstrates a lower pTau severity in the cerebellum, consistent with previous semi-quantitative studies [24, 25]. Indeed, the cerebellar pathological environment has been reported to exhibit a similar composition to that of very early AD in the temporal lobe with very low expression of pTau [26]. A key question is whether the cerebellum is a suitable region to use as pseudo-reference for comparison with the other brain areas in PET analysis. Clinical PET studies in AD have used the cerebellum as pseudo-reference region to quantify cerebral specific binding of the TSPO radiotracer [4, 8, 17]. The underlying assumption is that the cerebellum has lower binding that is not altered in the progression of the pathological condition [4, 17, 27]. Our *post-mortem* data demonstrate less AD pathology in the cerebellum associated with a lower and consistent TSPO expression over the course of the Braak stages, therefore, supporting the cerebellum as a reference region for TSPO binding.

Comparing the expression of microglial proteins showed disparity between the temporal lobe and cerebellum. Overall, the temporal lobe has higher expression of MSR-A, while the cerebellum has higher expression of HLA–DR. However, only Iba1 expression was significantly changed over the course of the disease with an increase in the cerebellum. Interestingly, TSPO was increased in the temporal lobe, with the highest expression between Braak stages III–IV and stages V–VI, but not in the cerebellum. This finding indicates that TSPO could represent late stage activation, potentially demonstrating a more reactive, phagocytic microglia [28], and that although microglia may be spatially redistributed in AD, the quantitative changes within the microglia may relate to their TSPO-labelled mitochondria. Of note, TSPO expression, as detected by the antibody, was observed in microglia and endothelial cells only. With microglia representing 10% [29, 30] and endothelial cells 0.3% [31] of the cerebral cells, our assessment of the staining mainly reflected the TSPO + microglia.

A link was also detected between pTau and TSPO in the temporal lobe, consistent with an imaging study showing associations between [^11^C]PBR28 (TSPO) and [^18^F]AV1451 (tau) PET ligands in MCI and AD patients, which was stronger in AD [32]. Furthermore, an examination of clinical progression of AD found that TSPO and tau PET together were the best predictors of disease progression and cognitive decline [33]. The relationship between TSPO and pTau was also reported in a preclinical study with the knockout of TSPO in a mouse model of AD associated with reduced amount of tau aggregates [34]. This implies a longitudinal relationship between tau and TSPO with the pathological protein associated with increased TSPO levels. TSPO expression as a microglial mitochondrial receptor could be a key feature of AD [35] as supported in the preclinical AD model 5XFAD mice, in which Aβ and pTau exposure induced metabolic dysfunction in microglia [36]. Hence, the increased TSPO in the temporal lobe in the late stage of the disease might reflect microglial dysfunction at the level of mitochondria in AD.

Of note, Iba1, a marker of microglial motility [18], was increased in the cerebellum, rather than in the temporal lobe. This indicates microglia sensing changes in the brain homeostasis, potentially linked to Aβ deposition (diffuse plaques), while in late stage (as observed in temporal lobe), microglia cluster around Aβ plaques as reported by us and others [5–7, 37]. MSR-A expression was not modified in either region over the course of the disease, but its expression was higher in the temporal cortex (i.e., in the region with the more severe pathology) consistent with this marker having a vulnerability to neurodegeneration [7].

The rs6971 polymorphism impacts the binding ability of TSPO radioligands, thus to observe whether the immunostaining of TSPO was dependent on the polymorphism, we genotyped the cases for the SNP. In our cohort, the TSPO genotype corresponding to high-affinity binders had the highest prevalence (42.59%), followed by the mixed-affinity binders (40.74%) and with the low-affinity binders the least prevalent (16.67%), as reported for European populations [23]. Also, the TSPO immunostaining was not affected by the TSPO polymorphism. This could be explained by the difference in the binding site on the TSPO protein, with the radioligand recognising nine amino acid residues across all five transmembrane domains [10], whereas the antibody binding is between amino acid bases 76 and 169.

The inflammatory microenvironment revealed an increase in IL15 in the temporal lobe over the course of the disease. IL15 is secreted by phagocytic cells to induce an immune response primarily from T cells and natural killer (NK) cells. Elevated IL15 levels in the CSF and serum of AD patients correlated with severity of cognitive dysfunction [38, 39], and with its expression associated with age of onset [38]. No differences were observed in the neuroinflammatory environment of the cerebellum, possibly as the result of a more homeostatic brain condition with lower AD pathology and microglial reactivity. The inflammatory status of the human brain presents a conflicting profile, with some studies reporting more inflammatory changes during aging than in AD [40]. However, our study confirms the importance of IL15 in AD, mainly in presence of severe pathology.

Two negative associations were found in the temporal lobe between the granulocyte–macrophage colony-stimulating factor (GM-CSF) with TSPO and the macrophage-derived chemokine (MDC/CCL22) with HLA–DR. GM-CSF is known to stimulate microglial growth and to be associated with reducing proinflammatory cells [41], with increased GM-CSF reported in the CSF of AD patients [42]. This inflammatory protein is currently being examined as a potential therapeutic target due to its ability to stimulate the innate immune system to clear the pathological proteins via microglial phagocytosis [41]. Of note, the negative GM-CSF association with TSPO and the TSPO relation to pTau is consistent with the hypothesis that microglial reactivity participates in pTau spreading [28], possibly due to dysfunctional microglial phagocytic activity as the result of mitochondrial damage in pathologically affected regions [43].

MDC/CCL22 acts on dendritic, natural killer (NK) and T cells to elicit an immune response [44]. T cell infiltration in in the human brain is a recognised feature of AD [5, 45]. HLA–DR, expressed by microglia/perivascular macrophages, is known to interact with T helper cells to initiate the production of antibodies. Interestingly in the temporal lobe, the negative association between MDC/CCL22 and HLA–DR, both required to activate T cells, implies an impaired control of T cell activation.

In the cerebellum, only one positive association was observed between IL16, a CD4 + cell chemoattractant (T cells, monocytes/macrophages), and Iba1. High levels of IL16 have been detected in blood plasma in early stages of AD but not in the later stages [46], consistent with our finding that the cerebellum exhibits early AD pathology. Interestingly, while overall all inflammatory markers were higher in the temporal lobe, four markers were more highly expressed in the cerebellum, namely, VEGF, IL8HA, IL10 and IL2 which may reflect the difference in the regional vulnerability for AD pathology.

## Conclusion

Our study supports the cerebellum as an appropriate region to be used as pseudo-reference for the TSPO PET studies, independently of the ligand binding. We also report that TSPO microglial expression appears to be associated with pTau and late stage of the disease, potentially highlighting a microglial profile associated with mitochondrial dysfunction which worsens with disease progression.

### Supplementary Information

**Additional file 1: Table S1.** Protein concentration for the Proinflammatory panel 1 per area and Braak stage. **Table S2.** Protein concentration for the Cytokine panel 1 per area and Braak stage. **Table S3.** Protein concentration for the Chemokine panel 1 per area and Braak stage. **Table S4.** Comparisons for protein concentration between temporal lobe and cerebellum. **Table S5.** Correlations between post-mortem delay and all markers in temporal lobe and cerebellum**.**

## Data Availability

The data analyzed during the current study are available from the corresponding author on reasonable request.

## Abbreviations

Aβ: Amyloid beta
AD: Alzheimer’s disease
APOE: Apolipoprotein E
Cb: Cerebellum
DAB: 3,3′-Diaminobenzidine
GM-CSF: Granulocyte–macrophage colony-stimulating factor
HLA–DR: Human leukocyte antigen–DR isotype
Iba1: Ionized calcium-binding adapter molecule 1
MDC: Macrophage-derived chemokine
MSD: MesoScale Discovery
MSR-A: Macrophage scavenging receptor-A
PBR: Peripheral benzodiazepine receptor
PET: Positron emission tomography
ROI: Region of interest
SNP: Single nucleotide polymorphism
TL: Temporal lobe
TSPO: Translocator protein
