# Supplementary material for: The mitochondrial protein TSPO in Alzheimer’s disease: relation to the severity of AD pathology and the neuroinflammatory environment
Source: J Neuroinflammation. 2023 Aug 14;20:186. doi: 10.1186/s12974-023-02869-9 (PMC10424356; doi:10.1186/s12974-023-02869-9)
Supplement: Supplementary file 1 — Additional file 1: Table S1. Protein concentration for the Proinflammatory panel 1 per area and Braak stage. Table S2. Protein concentration for the Cytokine panel 1 per area and Braak stage. Table S3. Protein concentration for the Chemokine panel 1 per area and Braak stage. Table S4. Comparisons for protein concentration between temporal lobe and cerebellum. Table S5. Correlations between post-mortem delay and all markers in temporal lobe and cerebellum. [file 12974_2023_2869_MOESM1_ESM.docx]

**The mitochondrial protein TSPO in Alzheimer’s disease: relation to the severity of AD pathology and the neuroinflammatory environment**

Emma F. Garland^1^, Oliver Dennett^1^, Laurie C. Lau^1^, Michel Bottlaender^2^, James A.R. Nicoll^1,3^, Delphine Boche^1^.

^1^Clinical Neurosciences, Clinical and Experimental Sciences, Faculty of Medicine, University of Southampton, Southampton, UK

^2^Paris-Sacaly University, CEA, CNRS, Inserm, BioMaps, Service Hospitalier Frederic Joliot F91400 Orsay, France

^3^Department of Cellular Pathology, University Hospital Southampton NHS Trust, Southampton, UK

**Supplementary Table 1**. Protein concentration for the Proinflammatory panel 1 per area and Braak stage

|  | Marker | Braak 0-II  (n=19) | Braak III-IV  (n=18) | Braak V-VI  (n=15) | P value |
| --- | --- | --- | --- | --- | --- |
| Temporal lobe | IFNγ | 0.8x10^-8^ [0.5x10^-8^ – 1.1x10^-8^] | 0.8 x10^-8^ [0.6x10^-8^ – 1.3x10^-8^] | 2.4x10^-8^ [0.5x10^-8^ – 0.9x10^-8^] | 0.241 |
|  | IL10 | 0.1x10^-8^ [0.09x10^-8^ – 0.2x10^-8^] | 0.2x10^-8^ [0.1x10^-8^ – 0.2x10^-8^] | 0.1 x10^-8^ [0.08x10^-8^ – 0.2x10^-8^] | 0.129 |
|  | IL12p70 | 0.5x10^-8^ [0.4x10^-8^ – 0.6x10^-8^] | 0.4 x10^-8^ [0.3x10^-8^ – 0.6x10^-8^] | 0.4 x10^-8^[0.3x10^-8^ – 0.5x10^-8^] | 0.847 |
|  | IL13 | 4.7x10^-8^ [3.7x10^-8^ – 5.9x10^-8^] | 5.0x10^-8^ [4.0x10^-8^ – 5.6x10^-8^] | 5.2 x10^-8^ [4.2x10^-8^ – 6.1x10^-8^] | 0.885 |
|  | IL1β | 2.6 x10^-8^ [1.6x10^-8^ – 6.0x10^-8^] | 3.1x10^-8^ [2.3x10^-8^ – 4.0x10^-8^] | 0.9 x10^-8^ [0.4x10^-8^ – 3.1x10^-8^] | 0.592 |
|  | IL2 | 0.3 x10^-8^ [0.2x10^-8^ – 0.4x10^-8^] | 0.3 x10^-8^ [0.3x10^-8^ – 0.4x10^-8^] | 0.2x10^-8^ [0.2x10^-8^ – 0.4x10^-8^] | 0.140 |
|  | IL4 | 0.2x10^-8^ [0.2x10^-8^ – 0.3x10^-8^] | 0.2x10^-8^ [0.2x10^-8^ – 0.3x10^-8^] | 0.2x10^-8^ [0.2x10^-8^ – 0.3x10^-8^] | 0.628 |
|  | IL6 | 5.1x10^-8^ [1.9x10^-8^ –19.9x10^-8^] | 8.3x10^-8^ [3.6x10^-8^ – 17.4x10^-8^] | 5.0x10^-8^ [2.7x10^-8^ – 14.7x10^-8^] | 0.708 |
|  | IL8 | 2.6x10^-7^[1.6x10^-7^ – 7.0x10^-7^] | 2.9x10^-7^ [1.8x10^-7^ – 8.6x10^-7^] | 2.3x10^-7^ [1.5x10^-7^ – 5.7x10^-7^] | 0.826 |
|  | TNFα | 0.6x10^-8^ [0.5x10^-8^ – 0.7x10^-8^] | 0.6x10^-8^ [0.4x10^-8^ – 0.8x10^-8^] | 0.5x10^-8^ [0.4x10^-8^ – 0.7x10^-8^] | 0.533 |
| Cerebellum | IFNγ | 3.6x10^-9^[4.5x10^-9^ – 2.9x10^-9^] | 4.7x10^-9^ [5.6x10^-9^ - 3.7x10^-9^] | 3.6x10^-9^[4.4x10^-9^ – 3.0x10^-9^] | 0.173 |
|  | IL10 | 1.8x10^-9^[2.1x10^-9^ – 1.2x10^-9^] | 1.9x10^-9^[2.6x10^-9^ – 1.7 x10^-9^] | 1.8 x10^-9^[2.0x10^-9^ – 1.6x10^-9^] | 0.265 |
|  | IL12p70 | 1.9x10^-9^[2.3x10^-9^ – 1.1x10^-9^] | 2.5x10^-9^[3.2x10^-9^ – 1.8x10^-9^] | 1.9x10^-9^[2.3x10^-9^ – 1.7x10^-9^] | 0.093 |
|  | IL13 | 4.1x10^-8^[5.9x10^-8^ – 3.5x10^-8^] | 4.2x10^-8^[6.7x10^-8^ – 2.8x10^-8^] | 3.8x10^-8^[5.3x10^-8^ – 3.0x10^-8^] | 0.545 |
|  | IL1β | 5.8x10^-8^[1.6x10^-8^ – 3.9x10^-9^] | 1.0x10^-8^[2.1x10^-8^ – 6.4x10^-8^] | 4.9x10^-9^[1.1x10^-8^ – 4.2x10^-9^] | 0.179 |
|  | IL2 | 4.6x10^-9^[5.4x10^-9^ – 3.5x10^-9^] | 5.5x10^-9^[6x10^-9^ – 4.5x10^-9^] | 4.7x10^-9^[5.4x10^-9^ – 4.1x10^-9^] | 0.161 |
|  | IL4 | 1.2x10^-9^[1.4x10^-9^ – 8.5x10^-10^] | 1.4x10^-9^[1.8x10^-9^ – 1.2x10^-9^] | 1.4x10^-9^[1.4x10^-9^ – 1.2x10^-9^] | 0.061 |
|  | IL6 | 5.1x10^-8^[9.6x10^-8^ – 2.2x10^-8^] | 1.4x10^-7^[2.9x10^-7^ – 2.8x10^-8^] | 2.5x10^-8^[1.3x10^-7^ – 2.0x10^-8^] | 0.256 |
|  | IL8 | 1.9x10^-7^[3.7x10^-7^ – 9.3 x10^-8^] | 1.4x10^-7^[5.6x10^-7^ – 9.3x10^-8^] | 1.9x10^-7^[3.4x10^-7^ – 2.1x10^-7^] | 0.845 |
|  | TNFα | 3.6x10^-9^[4.0x10^-9^ – 2.3 x10^-9^] | 3.5 x10^-9^[4.9x10^-9^ – 2.8 x10^-9^] | 3.3 x10^-9^[3.7 x10^-9^ – 2.9 x10^-9^] | 0.408 |

P values obtained using Kruskal-Wallis’ test with significant P value (<0.05) in bold

Data displayed as - median [interquartile range]

**Supplementary Table 2**. Protein concentration for the Cytokine panel 1 per area and Braak stage

|  | Marker | Braak 0-II  (n=19) | Braak III-IV  (n=18) | Braak V-VI  (n=15) | P value |
| --- | --- | --- | --- | --- | --- |
| Temporal lobe | GM-CSF | 0.1x10^-8^ [0.1x10^-8^ – 0.2x10^-8^] | 0.2x10^-8^ [0.1x10^-8^ – 0.3x10^-8^] | 0.1x10^-8^ [0.07x10^-8^ – 0.2x10^-8^] | 0.254 |
|  | IL1α | 4.0x10^-8^ [2.8x10^-8^ – 6.2x10^-8^] | 5.7x10^-8^ [3.9x10^-8^ – 8.4x10^-8^] | 3.0x10^-8^ [1.9x10^-8^ – 11.8x10^-8^] | 0.424 |
|  | IL12/IL23p70 | 1.3x10^-8^ [1.1x10^-8^ – 1.9x10^-8^] | 1.6x10^-8^ [1.1x10^-8^ – 2.0x10^-8^] | 1.2x10^-8^ [1.0x10^-8^ – 1.8x10^-8^] | 0.455 |
|  | **IL15** | **6.7x10^-8^ [6.0x10^-8^ – 8.1x10^-8^]** | **7.5x10^-8^ [6.4x10^-8^ – 9.5x10^-8^]** | **9.3x10^-8^ [7.6x10^-8^ – 10.7x10^-8^]** | **0.019*** |
|  | IL16 | 4.2x10^-6^ [3.9x10^-6^ – 6.9x10^-6^] | 6.0x10^-6^ [3.7x10^-6^ – 8.4x10^-6^] | 6.3x10^-6^ [4.3x10^-6^ – 7.7x10^-6^] | 0.586 |
|  | IL17A | 1.3x10^-8^ [1.1x10^-8^ – 1.7x10^-8^] | 1.5x10^-8^ [0.9x10^-8^ – 1.8x10^-8^] | 1.7x10^-8^ [1.3x10^-8^ – 1.9x10^-8^] | 0.192 |
|  | IL5 | 0.08x10^-8^ [0.05x10^-8^ – 0.2x10^-8^] | 0.2x10^-8^ [0.09x10^-8^ – 0.2x10^-8^] | 0.09x10^-8^ [0.05x10^-8^ – 0.2x10^-8^] | 0.188 |
|  | IL7 | 0.4x10^-8^ [0.3x10^-8^ – 0.6x10^-8^] | 0.6x10^-8^ [0.4x10^-8^ –0.8 x10^-8^] | 0.4x10^-8^ [0.2x10^-8^ – 0.5x10^-8^] | 0.070 |
|  | TNFβ | 0.1x10^-8^ [0.09x10^-8^ – 0.2x10^-8^] | 0.1x10^-8^ [0.1x10^-8^ – 0.2x10^-8^] | 0.1x10^-8^ [0.1x10^-8^ – 0.2x10^-8^] | 0.916 |
|  | VEGF | 2.0x10^-8^ [1.0x10^-8^ – 3.7x10^-8^] | 2.5x10^-8^ [1.2x10^-8^ – 4.9x10^-8^] | 2.2x10^-8^ [0.7x10^-8^ – 4.1x10^-8^] | 0.797 |
| Cerebellum | GM-CSF | 5.9x10^-10^[7.4x10^-10^ – 2.8x10^-10^] | 4.6x10^-10^ [6.7x10^-10^ – 3.1x10^-10^] | 2.7x10^-10^[4.2x10^-10^–2.5x10^-10^] | 0.447 |
|  | IL1α | 5.5x10^-9^[6.8x10^-9^ – 4.4x10^-9^] | 6.6x10^-9^[1.4x10^-8^ – 4.7x10^-9^] | 9.5x10^-9^[1.2x10^-9^ – 4.6x10^-9^] | 0.291 |
|  | IL12/IL23p70 | 6.9x10^-9^[1.6x10^-8^ – 3.6x10^-9^] | 1.1x10^-8^[1.2x10^-8^ – 7.3x10^-9^] | 1.0x10^-9^[1.3x10^-8^ – 5.9x10^-9^] | 0.746 |
|  | IL15 | 5.8x10^-8^[6.3x10^-8^ – 5.2x10^-8^] | 5.8x10^-8^[6.4x10^-8^ – 4.7x10^-8^] | 6.3x10^-8^[5.5x10^-8^ – 7.2x10^-8^] | 0.486 |
|  | IL16 | 2.6x10^-6^[4.6x10^-6^ – 1.8x10^-6^] | 4.7x10^-6^[6.3x10^-6^ – 3.1x10^-6^] | 5.0x10^-6^[5.7x10^-6^ – 3.3x10^-6^] | 0.060 |
|  | IL17A | 1.5x10^-8^[1.7x10^-8^ – 1.1x10^-8^] | 1.2x10^-8^[1.6x10^-8^ – 1.1x10^-8^] | 1.4x10^-8^[1.5x10^-8^ – 9.5x10^-9^] | 0.700 |
|  | IL5 | 4.8x10^-10^[8.6x10^-10^ – 2.9x10^-10^] | 5.9x10^-10^[7.9x10^-10^ – 3.88x10^-10^] | 6.8x10^-10^[8.6x10^-10^ – 4.4x10^-10^] | 0.356 |
|  | IL7 | 5.2x10^-10^[6.5x10^-10^ – 3.9x10^-10^] | 5.8x10^-10^[8.6x10^-10^ – 2.11x10^-10^] | 5.4x10^-10^[7.0x10^-10^ – 4.6x10^-10^] | 0.996 |
|  | TNFβ | 4.8x10^-10^[5.3x10^-10^ – 3.0x10^-10^] | 3.4x10^-10^[5.22x10^-10^ – 2.0x10^-10^] | 3.1x10^-10^[4.4x10^-10^ – 2.3x10^-10^] | 0.513 |
|  | VEGF | 1.4x10^-7^[2.3x10^-7^ – 5.0x10^-8^] | 9.6x10^-8^[2.4x10^-7^ – 2.8x10^-8^] | 7.4x10^-8^[1.7x10^-7^ – 5.4x10^-8^] | 0.750 |

P values obtained using Kruskal-Wallis’ test with significant P value (<0.05) in bold

Data displayed as - median [interquartile range]

**Supplementary Table 3.** Protein concentration for the Chemokine panel 1 per area and Braak stage

|  | Marker | Braak 0-II  (n=19) | Braak III-IV  (n=18) | Braak V-VI (n=15) | P value |
| --- | --- | --- | --- | --- | --- |
| Temporal lobe | Eotaxin | 1.0x10^-7^ [0.6x10^-7^ – 1.7x10^-7^] | 1.1x10^-7^ [0.7x10^-7^ – 1.5x10^-7^] | 1.1x10^-7^ [0.8x10^-7^ – 2.5x10^-7^] | 0.510 |
|  | Eotaxin-3 | 2.1x10^-7^ [1.5x10^-7^ – 3.6x10^-7^] | 5.1x10^-7^ [2.1x10^-7^ – 7.8x10^-7^] | 3.2x10^-7^ [1.5x10^-7^ – 5.5x10^-7^] | 0.105 |
|  | IL8 (HA) | 1.5x10^-6^ [1.1x10^-6^ – 2.9x10^-6^] | 1.9x10^-6^ [0.8x10^-6^ – 2.9x10^-6^] | 1.7x10^-6^ [1.1x10^-6^ – 4.8x10^-6^] | 0.924 |
|  | IP10 | 2.7x10^-7^ [1.7x10^-7^ – 4.3x10^-7^] | 4.3x10^-7^ [2.3x10^-7^ – 12.3x10^-7^] | 4.7x10^-7^ [3.0x10^-7^ – 7.3x10^-7^] | 0.314 |
|  | MCP1 | 6.0x10^-7^ [3.3x10^-7^ – 10.3x10^-7^] | 7.3x10^-7^ [4.4x10^-7^ – 14.1x10^-7^] | 11.3x10^-7^ [7.3x10^-7^ – 16.3x10^-7^] | 0.323 |
|  | MCP4 | 2.0x10^-7^ [1.6x10^-7^ – 2.5x10^-7^] | 1.7x10^-7^ [1.6x10^-7^ – 2.5x10^-7^] | 2.4x10^-7^ [1.6x10^-7^ – 2.5x10^-7^] | 0.084 |
|  | MDC | 2.7x10^-7^ [1.9x10^-7^ – 3.6x10^-7^] | 2.7 x10^-7^ [1.4x10^-7^ – 3.8x10^-7^] | 3.3 x10^-7^ [2.7x10^-7^ – 8.9x10^-7^] | 0.174 |
|  | MIP1α | 1.3x10^-7^ [1.0x10^-7^ – 1.6x10^-7^] | 1.7x10^-7^ [1.0x10^-7^ – 3.0x10^-7^] | 1.5x10^-7^ [1.3x10^-7^ – 2.9x10^-7^] | 0.235 |
|  | MIP1β | 1.9 x10^-7^ [1.7x10^-7^ – 2.3x10^-7^] | 2.6 x10^-7^ [1.6x10^-7^ – 4.2x10^-7^] | 1.8 x10^-7^ [1.7x10^-7^ – 3.2x10^-7^] | 0.242 |
|  | TARC | 2.2x10^-8^ [0.9x10^-8^ – 3.6x10^-8^] | 2.9x10^-8^ [2.1x10^-8^ – 4.7x10^-8^] | 2.4x10^-8^ [1.2x10^-8^ – 4.1x10^-8^] | 0.393 |
|  | Eotaxin | 1.9x10^-9^[2.4x10^-9^ – 1.5x10^-9^] | 1.8x10^-9^[2.5x10^-9^ – 1.6x10^-9^] | 2.2x10^-9^[2.5x10^-9^ – 1.6x10^-9^] | 0.965 |
| Cerebellum | Eotaxin 3 | 1.5x10^-7^[3.5x10^-7^ – 8.3x10^-8^] | 1.1x10^-7^[1.2x10^-7^ – 9.6x10^-8^] | 2.2x10^-7^[3.7x10^-7^ – 9.2x10^-8^] | 0.470 |
|  | IL8 (HA) | 5.7x10^-6^[8.4x10^-6^ – 4.8x10^-6^] | 5.7x10^-6^[7.6x10^-6^ – 4.8x10^-6^] | 7.2x10^-6^[9.3x10^-6^ –3.7x10^-6^] | 0.912 |
|  | IP10 | 2.1x10^-7^[3.1x10^-7^ – 1.2x10^-7^] | 1.4x10^-7^[3.5x10^-7^ – 1.1x10^-7^] | 2.3x10^-7^[5.0x10^-7^ – 10.0x10^-8^] | 0.734 |
|  | MCP1 | 2.3x10^-7^[4.3x10^-7^ – 1.5x10^-7^] | 2.3x10^-7^[1.4x10^-6^ – 1.3x10^-7^] | 2.7x10^-7^[4.0x10^-7^ – 1.9x10^-7^] | 0.913 |
|  | MCP4 | 1.5x10^-7^[2.0x10^-7^ – 1.0x10^-7^] | 1.5x10^-7^[1.8x10^-7^ – 1.2x10^-7^] | 1.2x10^-7^[1.9x10^-7^ – 1.1x10^-7^] | 0.861 |
|  | MDC | 2.4x10^-7^[3.4x10^-7^ – 1.5x10^-7^] | 1.6x10^-7^[2.7x10^-7^ – 1.1x10^-7^] | 2.1x10^-7^[3.9x10^-7^ – 1.2x10^-7^] | 0.487 |
|  | MIP1α | 7.0x10^-8^[8.8x10^-8^ – 5.5x10^-8^] | 6.8x10^-8^[1.3x10^-7^ – 6.1x10^-8^] | 6.9x10^-8^[1.0x10^-7^ – 5.1x10^-8^] | 0.625 |
|  | MIP1β | 9.9x10^-8^[1.1x10^-7^ – 7.1x10^-8^] | 9.0x10^-8^[1.4x10^-7^ – 7.3x10^-8^] | 7.1x10^-8^[9.6x10^-8^ – 6.5x10^-8^] | 0.352 |
|  | TARC | 2.8x10^-8^[3.3x10^-8^ – 1.6x10^-8^] | 2.5x10^-8^[3.3x10^-8^ – 1.6x10^-8^] | 2.2x10^-8^[2.8x10^-8^ – 1.7x10^-8^] | 0.744 |

P values obtained using Kruskal-Wallis test with significant P value (<0.05) in bold

Data displayed as - median [interquartile range]

**Supplementary Table 4.** Comparisons for protein concentration between temporal lobe and cerebellum

| Marker | Temporal lobe | Cerebellum | P value |
| --- | --- | --- | --- |
| **GM-CSF** | **1.387x10^-9^ [9.458x10^-10^-1.804x10^-9^]** | **4.240x10^-10^ [2.465x10^-10^-7.143x10^-10^]** | **<0.0001** |
| **IL1α** | **4.184x10^-8^ [2.660x10^-8^-8.473x10^-8^]** | **5.971x10^-9^ [4.607x10^-9^-1.163x10^-8^]** | **<0.0001** |
| **IL12/IL23p70** | **1.468e-008 [1.055e-008-1.781e-008]** | **9.353e-009 [5.434e-009-1.386e-008]** | **<0.0001** |
| **IL15** | **7.555x10^-8^ [6.237x10^-8^-9.360x10^-8^]** | **5.838x10^-8^ [5.054x10^-8^-6.649x10^-8^]** | **<0.0001** |
| **IL16** | **5.706x10^-6^ [3.960x10^-6^-7.814x10^-6^]** | **4.308x10^-6^ [2.067x10^-6^-5.509x10^-6^]** | **0.002** |
| IL17A | 1.449x10^-8^ [1.095x10^-8^-1.775x10^-8^] | 1.368x10^-8^ [1.060x10^-8^-1.715x10^-8^] | 0.607 |
| **IL5** | **1.084x10^-9^ [6.708x10^-10^-1.868x10^-9^]** | **5.870x10^-10^ [3.850x10^-10^-8.510x10^-10^]** | **<0.0001** |
| **IL7** | **4.284x10^-9^ [2.887x10^-9^-6.229x10^-9^]** | **5.740x10^-10^ [3.070x10^-10^-8.065x10^-10^]** | **<0.0001** |
| **TNFβ** | **1.346x10^-9^ [9.238x10^-10^-1.686x10^-9^]** | **3.470x10^-10^ [2.240x10^-10^-5.130x10^-10^]** | **<0.0001** |
| **VEGF** | **2.112x10^-8^ [1.119x10^-8^-4.035x10^-8^]** | **9.305x10^-8^ [4.297x10^-8^-2.316x10^-7^]** | **<0.0001** |
| **Eotaxin** | **1.115x10^-7^ [6.801x10^-8^-1.767x10^-7^]** | **1.885x10^-8^ [1.594x10^-8^-2.578x10^-8^]** | **<0.0001** |
| **Eotaxin 3** | **2.889x10^-7^ [1.529x10^-7^-5.623x10^-7^]** | **1.245x10^-7^ [9.198x10^-8^-2.551x10^-7^]** | **<0.0001** |
| **IL8 (HA)** | **1.660x10^-6^ [1.093x10^-6^-2.921x10^-6^]** | **5.730x10^-6^ [4.496x10^-6^-8.492x10^-6^]** | **<0.0001** |
| **IP10** | **4.065x10^-7^ [1.912x10^-7^-7.324x10^-7^]** | **1.929x10^-7^ [1.073x10^-7^-4.337x10^-7^]** | **0.0007** |
| **MCP1** | **7.915x10^-7^ [4.382x10^-7^-1.468x^10-6^]** | **2.311x10^-7^ [1.393x10^-7^-4.354x10^-7^]** | **<0.0001** |
| **MCP4** | **2.036x10^-7^ [1.631x10^-7^-2.670x^10-7^]** | **1.477x10^-7^ [1.075x10^-7^-1.936x10^-7^]** | **0.0001** |
| **MDC** | **2.787x10^-7^ [1.989x10^-7^-4.191x^10-7^]** | **2.052x10^-7^ [1.211x10^-7^-3.486x10^-7^]** | **0.012** |
| **MIP1α** | **1.455x10^-7^ [1.092x10^-7^-2.208x^10-7^]** | **6.897x10^-8^ [5.467x10^-8^-1.007x10^-7^]** | **<0.0001** |
| **MIP1β** | **1.973x10^-7^ [1.683x10^-7^-3.086x10^-7^]** | **8.807x10^-8^ [6.698x10^-8^-1.171x10^-7^]** | **<0.0001** |
| TARC | 2.706x10^-8^ [1.248x10^-8^-4.382x10^-8^] | 2.422x10^-8^ [1.569x10^-8^-3.234x10^-8^] | 0.957 |
| **IFNγ** | **7.872x10^-9^ [5.070x10^-9^-1.064x10^-8^]** | **3.941x10^-9^ [2.936x10^-9^-5.142x10^-9^]** | **<0.0001** |
| **IL1b** | **2.728x10^-8^ [1.786x10^-8^-4.660x10^-8^]** | **7.101x10^-9^ [4.149x10^-9^-1.846x10^-8^]** | **<0.0001** |
| **IL10** | **1.230x10^-9^ [9.295x10^-10^-1.536x10^-9^]** | **1.861x10^-9^ [1.490x10^-9^-2.279x10^-9^]** | **<0.0001** |
| **IL12p70** | **4.220x10^-9^ [3.027x10^-9^-5.198x10^-9^]** | **1.990x10^-9^ [1.484x10^-9^-2.597x10^-9^]** | **<0.0001** |
| IL13 | 4.988x10^-8^ [3.927x10^-8^-5.827x10^-8^] | 4.117x10^-8^ [3.240x10^-8^-5.717x10^-8^] | 0.097 |
| **IL2** | **2.901x10^-9^ [2.237x10^-9^-3.830x10^-9^]** | **4.905x10^-9^ [3.915x10^-9^-5.640x10^-9^]** | **<0.0001** |
| **IL4** | **2.134x10^-9^ [1.676x10^-9^-2.841x10^-9^]** | **1.342x10^-9^ [1.140x10^-9^-1.564x10^-9^]** | **<0.0001** |
| IL6 | 6.334x10^-8^ [2.594x10^-8^-1.506x10^-7^] | 5.288x10^-8^ [2.046x10^-8^-1.781x10^-7^] | 0.644 |
| **IL8** | **2.589x10^-7^ [1.647x10^-7^-6.947x10^-7^]** | **1.856x10^-7^ [9.305x10^-8^-4.485x10^-7^]** | **0.037** |
| **TNFα** | **5.854x10^-9^ [4.639x10^-9^-6.918x10^-9^]** | **3.423x10^-9^ [2.507x10^-9^-4.171x10^-9^]** | **<0.0001** |

P values obtained using Mann-Whitney U test with significant P value (<0.05) in bold

Data displayed as - median [interquartile range]

**Supplementary Table 5.** Correlations between post-mortem delay and all markers in temporal lobe and cerebellum

|  | Marker | R_s_ value | P value |
| --- | --- | --- | --- |
| Temporal lobe | Aβ | -0.165 | 0.215 |
|  | pTau | -0.223 | 0.098 |
|  | TSPO | -0.093 | 0.485 |
|  | Iba1 | -0.076 | 0.570 |
|  | HLA-DR | 0.155 | 0.267 |
|  | MSR-A | 0.060 | 0.649 |
| Cerebellum | Aβ | -0.063 | 0.643 |
|  | pTau | -0.250 | 0.063 |
|  | TSPO | -0.085 | 0.533 |
|  | Iba1 | -0.119 | 0.383 |
|  | HLA-DR | 0.123 | 0.372 |
|  | MSR-A | 0.009 | 0.946 |
